# Vision-Based Autonomous Quadrupedal Robot for Rapid Post-Earthquake Crack-Based Building Damage Detection

**DOI:** 10.3390/s26154977

**Published:** 2026-08-06

**Authors:** Kemal Hacıefendioğlu, Murat Günaydın, Ayşecan Bostan, Ahmet Can Altunışık

**Affiliations:** 1 Department of Civil Engineering, Faculty of Engineering, Karadeniz Technical University, 61080 Trabzon, Türkiye; muratgunaydin@ktu.edu.tr (M.G.); aysecanbostan61@gmail.com (A.B.); ahmetcan@ktu.edu.tr (A.C.A.); 2 Earthquake and Structural Health Monitoring Research Center, Karadeniz Technical University, 61080 Trabzon, Türkiye

**Keywords:** autonomous robotics, damage detection, post-earthquake field investigation, quadrupedal robot, YOLOv8n

## Abstract

Rapid and reliable crack-based visual damage detection after earthquakes is crucial for safe and effective disaster response. Manual inspections are often slow and hazardous for engineers in unstable structures. This study proposes a quadrupedal robotic inspection system for rapid post-earthquake crack-based visual damage detection in reinforced concrete structures. A Unitree Go2 robot equipped with an Intel RealSense D435i RGB-D camera collected a dataset of 3255 annotated crack images from both field and public sources. The YOLOv8n model, trained and deployed on an NVIDIA Jetson AGX Xavier, demonstrated high detection performance in laboratory tests on reinforced concrete specimens, with precision, recall, and mAP@50 values all exceeding 85%. The system provides fast, accurate, and automated structural health assessments, reducing human risk and improving inspection efficiency in hazardous post-disaster environments. Future work will focus on expanding damage detection capabilities and real-world deployment.

## 1. Introduction

Earthquakes pose a persistent threat to human life and built infrastructure, necessitating rapid, accurate, and efficient post-event structural damage assessments. Timely evaluations are critical for informing emergency responses, facilitating safe reoccupation, and prioritizing repair efforts. However, traditional assessment methods—primarily reliant on manual inspections—are often time-consuming, resource-intensive, and hazardous for engineers, particularly in buildings with slight or moderate damage levels [1,2].

Recent advancements in artificial intelligence (AI) and robotics have begun to transform post-earthquake structural evaluation practices. AI-based damage detection systems, especially those utilizing deep learning, have significantly improved image-based structural analysis tasks, such as crack detection, spalling classification, and severity estimation [3,4,5,6,7]. Convolutional neural networks (CNNs), generative adversarial networks (GANs), and transfer learning approaches have enhanced model accuracy and generalization capabilities even when training data is limited [8,9,10]. Moreover, hybrid models incorporating attention mechanisms or transformer architectures have further advanced performance in multimodal damage detection scenarios [11].

Complementing these AI-driven approaches, robotic systems offer reliable and scalable solutions for data collection in hazardous environments. Unmanned aerial vehicles (UAVs), equipped with high-resolution imaging sensors, have become crucial tools for surveying extensive damage in remote or difficult-to-access areas [12,13]. Similarly, autonomous ground vehicles (AGVs), notably quadrupedal robotic platforms equipped with intelligent sensors and real-time navigation capabilities, have proven effective in confined or debris-laden environments [14,15,16]. These robotic platforms can effectively gather crucial visual and geometric data, significantly reducing human exposure to hazardous conditions.

Despite these advancements, substantial challenges persist. Deep learning models typically require extensive labeled datasets, yet acquiring comprehensive and high-quality data from real post-disaster scenarios remains challenging and costly [17,18]. Computational limitations also restrict the deployment of advanced models in resource-limited environments. Furthermore, the “black-box” nature of many deep learning architectures raises issues regarding interpretability, reliability, and trustworthiness in critical decision-making contexts [12,19].

Addressing these challenges, this study proposes an autonomous quadrupedal robotic system integrating deep learning for post-earthquake damage detection through structured laboratory-based damage simulations. A custom dataset comprising 3255 annotated crack images sourced from the Roboflow platform, along with additional images of shear and bending cracks and spalling damage from reinforced concrete (RC) specimens, was utilized to train a YOLOv8n object detection model. The trained model was deployed on a Unitree Go2 robot, equipped with an Intel RealSense D435i camera and an NVIDIA Jetson AGX Xavier edge computing device. This research underscores the potential of integrating computer vision and mobile robotics technologies to substantially enhance the speed, safety, and precision of post-earthquake structural damage assessments. By overcoming limitations inherent in traditional inspection methods and uniting cutting-edge technologies from robotics and AI, this work aims to significantly advance structural damage assessment methodologies and contribute to improved disaster response and recovery strategies.

The main contributions of this study can be summarized as follows:

- A quadrupedal robotic inspection framework is developed by integrating the Unitree Go2 platform, an Intel RealSense D435i RGB-D camera, ROS 2 middleware, and onboard edge computing for crack-based post-earthquake visual damage inspection.
- A YOLOv8-based object detection model is trained and deployed for rapid surface crack detection using a dataset consisting of field and publicly available crack images, enabling automated localization of visible damage indicators on reinforced concrete surfaces.
- Unlike UAV-based approaches that are mainly suitable for external or aerial inspection, the proposed quadrupedal platform is intended for close-range inspection in ground-level, confined, or partially obstructed environments where human access may be unsafe after an earthquake.
- The study demonstrates an end-to-end robotic vision pipeline, including image acquisition, onboard inference, detection visualization, and pose-supported mapping of detected crack regions.
- The proposed system is experimentally validated on a reinforced concrete laboratory specimen, providing an initial controlled demonstration of robotic crack detection for post-earthquake inspection scenarios.
- The limitations of the current system are explicitly recognized, particularly that the present model focuses on visible surface cracks and does not yet distinguish between cosmetic defects and structurally critical damage without further engineering assessment.

## 2. Research Framework

**Figure 1:**
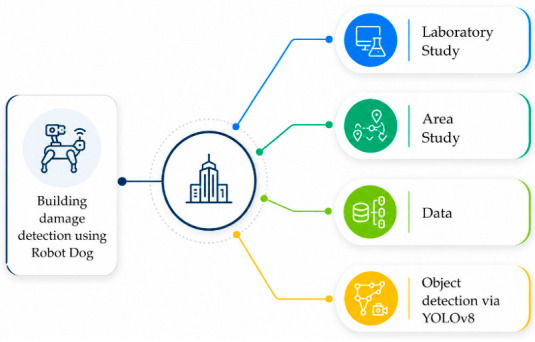
Framework of the study.

Figure 1 presents the general framework of the study, which enables a real-time, efficient, and accurate crack-based visual damage detection procedure following an earthquake. For this purpose, the data were collected from laboratory tests and databases containing records of earthquake-induced damage from earthquake-prone areas. The laboratory phase was specifically designed to validate the core performance parameters of the proposed model and robotic system, using detailed measurements conducted on test specimens featuring artificial cracks and simulated damage scenarios. The gathered visual data are then annotated and processed using a YOLOv8-based deep learning model for automated crack-based visual damage detection.

### 2.1. Dataset and Training Procedure

**Figure 2:**
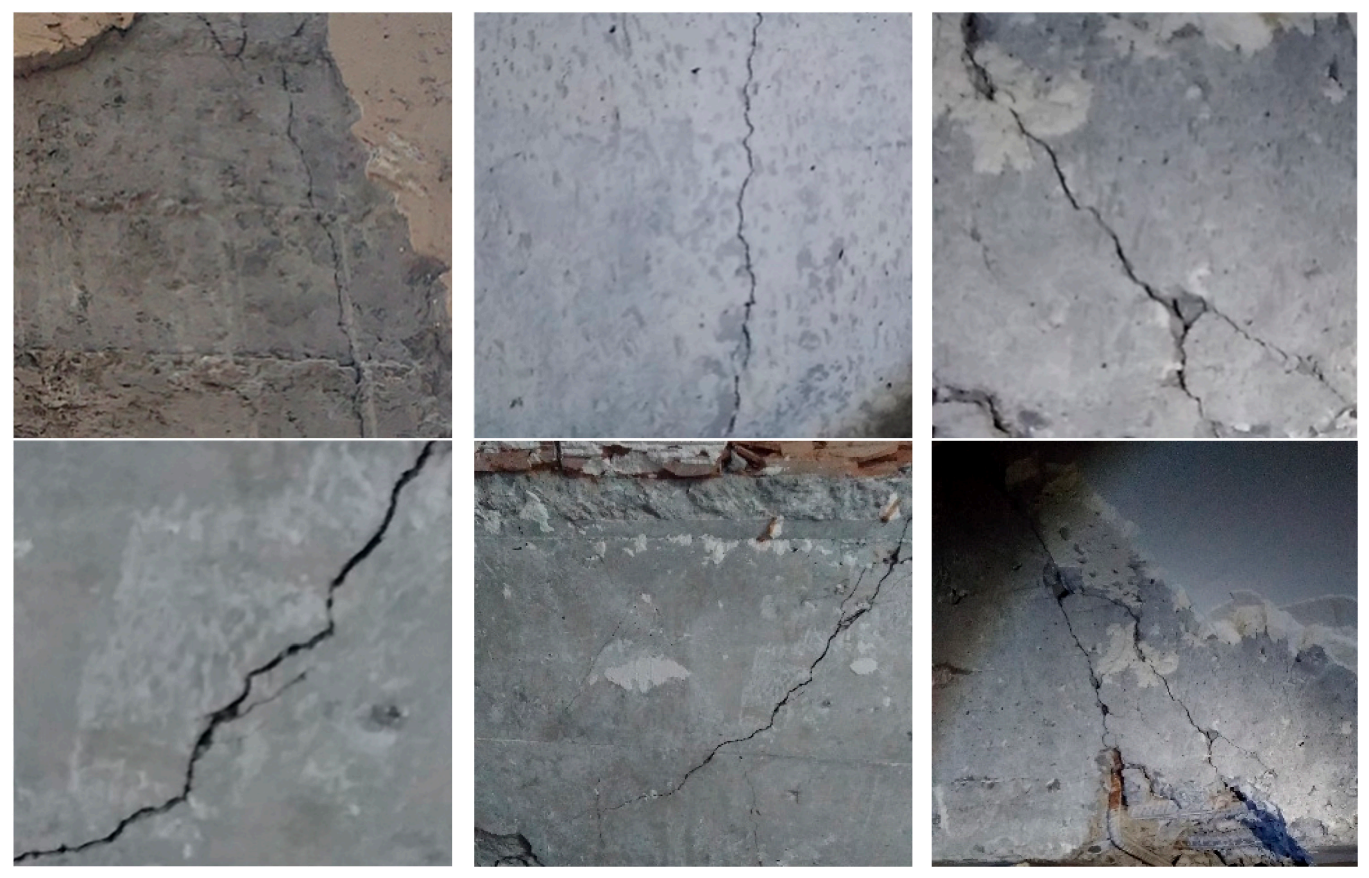
Some crack images captured during field investigations of the Kahramanmaraş earthquakes.

The image dataset used in this study was constructed from complementary data sources in order to improve the diversity and practical relevance of the crack detection model. The first source consisted of publicly available crack images obtained through the Roboflow platform. The second source included crack images captured during field investigation studies conducted after the Kahramanmaraş earthquakes. Representative examples of these field images are shown in Figure 2. These images were collected from reinforced concrete structural elements, including columns, beams, and shear walls, and contained visible earthquake-related surface crack patterns.

Roboflow was used as a dataset management and annotation platform because it provides an integrated environment for organizing images, creating labels, and exporting annotations in formats compatible with object detection models. All images obtained from Roboflow and the field investigations were combined and annotated in YOLO format. Each annotation file included the crack class ID and the normalized bounding-box coordinates corresponding to the visible crack region.

The overall dataset consisted of 3255 annotated crack images. In order to clearly separate model development from independent testing, the data were organized into training, validation, and test subsets. The training subset included 2658 labeled images and was used for learning the model parameters. The validation subset consisted of 300 images and was used to monitor training behavior, support model selection, and reduce the risk of overfitting. These two subsets were used only during the model development stage.

The test subset consisted of 297 images and was kept independent from the training and validation process. In this study, the final test evaluation was performed using images obtained from reinforced concrete laboratory specimens during the robotic inspection experiments. Therefore, the test stage represents an independent laboratory-based validation of the trained YOLOv8n model on a physical specimen rather than a subset used during model optimization. This strategy was adopted to provide a more realistic assessment of the model performance when integrated with the Unitree Go2 quadrupedal robotic inspection platform.

**Figure 3:**
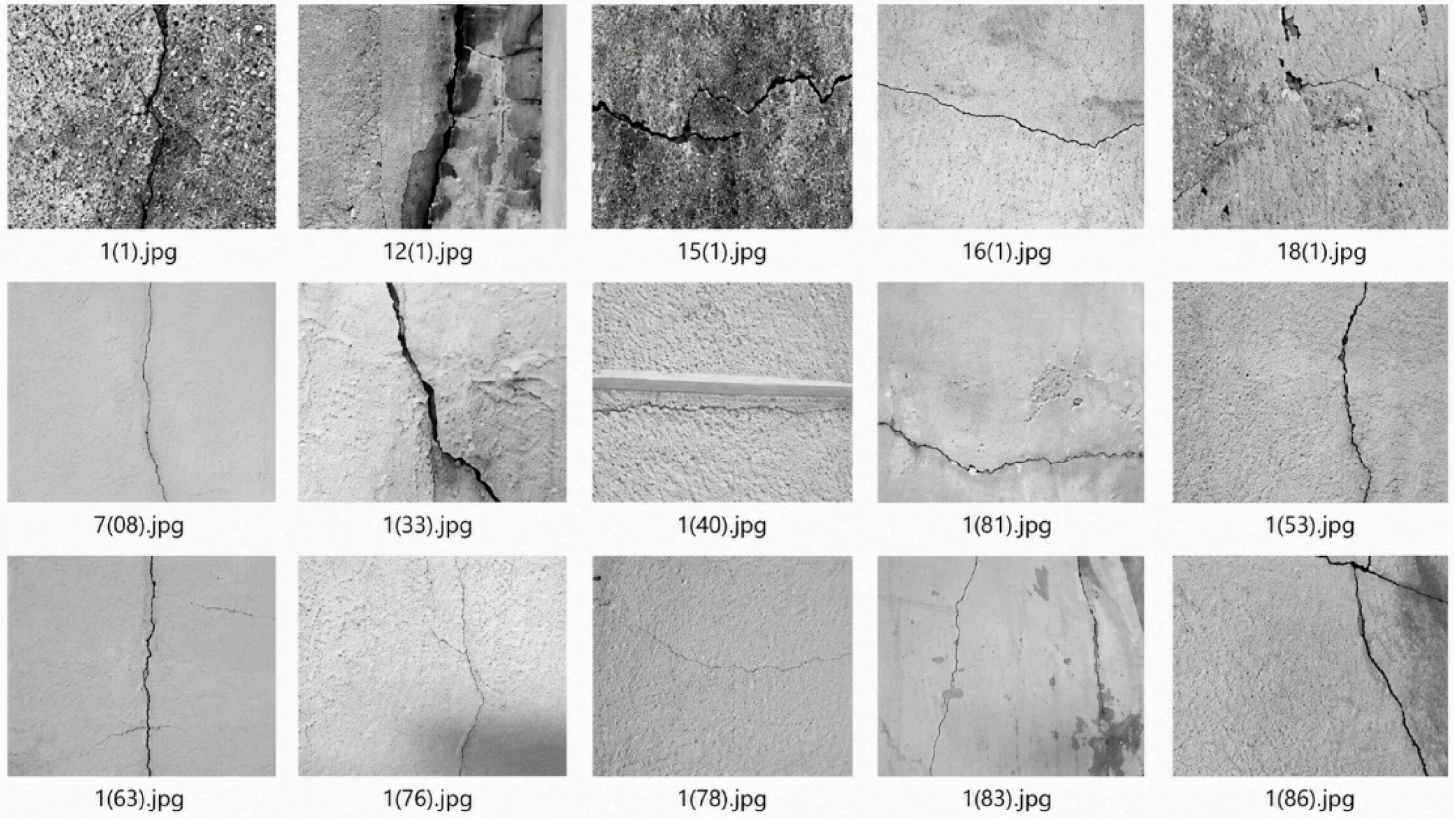
Sample images from the dataset.

**Figure 4:**
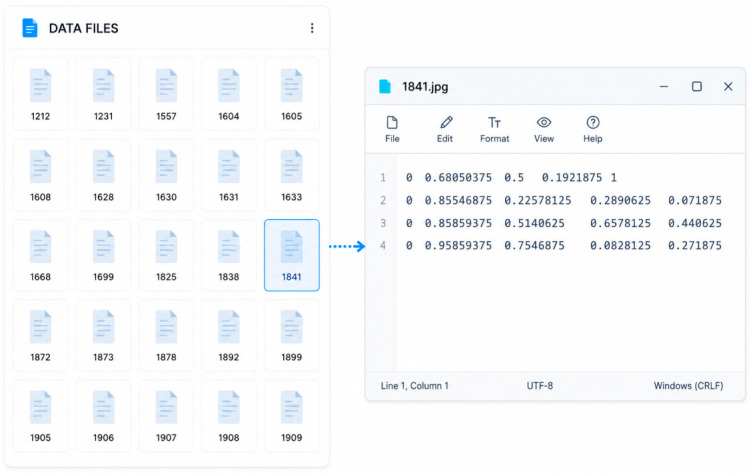
Label of files for the crack images.

Sample images and corresponding label files from the dataset are presented in Figure 3 and Figure 4. The labeling format follows the YOLO convention, including the class ID of each crack, the normalized center coordinates (x,y), and the normalized width (w) and height (h) of the bounding box. Normalizing the bounding-box coordinates with respect to image dimensions enables the model to process images with different resolutions and sizes.

In addition, the dataset file structure was organized according to YOLO training requirements. Each image was paired with a corresponding .txt annotation file within a consistent directory structure. Data preprocessing and augmentation techniques, including rotation, flipping, and brightness adjustment, were applied during training to improve robustness against variations in lighting conditions, viewing angles, crack orientation, and surface texture. These procedures were intended to increase the generalization capability of the model for crack-based visual damage detection.

**Figure 5:**
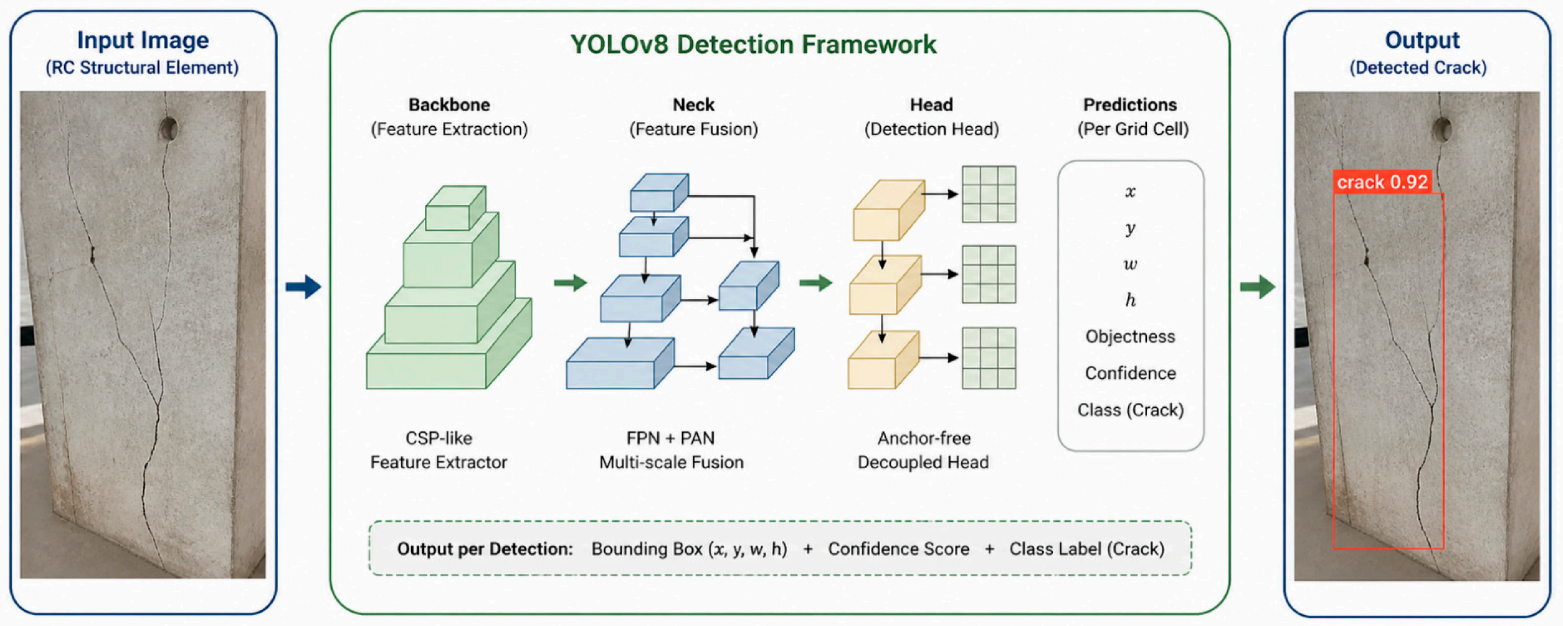
Schematic representation of the YOLO-based crack detection workflow.

The general detection workflow of the YOLO-based model is illustrated in Figure 5 using an author-prepared schematic representation. In this workflow, the input image is processed through feature extraction and multi-scale feature fusion stages, followed by a detection head that predicts bounding-box coordinates, confidence scores, and the crack class label. This simplified representation is provided to explain the detection pipeline used in the present study and is based on the YOLO literature and official YOLOv8n technical documentation [20].

In a YOLO-based network, an input image I with dimensions (*H*, *W*) is processed to produce an output that includes the bounding box parameters *B* = (*x*, *y*, *w*, *h*), a confidence score (*C*), and a class label (γ) for each detected damage. The localization loss *L_box_* quantifies the error in predicting the bounding box’s position and size. It is calculated by comparing the predicted coordinates and dimensions (*x*, *y*, *w*, *h*) with the ground truth values (x*, y*, w*, h*). The loss function is defined in Equation (1) as:

(1) $$L_{box}=\alpha \left[(x-x^{*}{)}^{2}+(y-y^{*}{)}^{2}\right]+\beta \left[(\sqrt{w}-\sqrt{w^{*}}{)}^{2}+(\sqrt{h}-\sqrt{h^{*}}{)}^{2}\right]$$

Here, *α* and *β* are weighting coefficients that balance the contribution of position and size errors, and the square root transformation helps stabilize the learning of bounding box dimensions, especially for objects of varying scales.

The confidence loss *L_conf_* quantifies how accurately the model predicts the presence or absence of an object (e.g., a crack) in a given region. It is typically computed using logistic regression or binary cross-entropy, comparing the predicted probability $p_{i}$ with the ground truth label $p_{i}^{*}$, where $p_{i}^{*}$ = 1 if an object is present in region *i* and $p_{i}^{*}$ = 0 otherwise [21]. The loss is calculated in Equation (2) as follows:

(2) $$L_{conf}=-\sum_{i=1}^{N}\left[p_{i}^{*}log(p_{i})+(1-p_{i}^{*})log(1-p_{i})\right]$$

Here, *N* is the total number of candidate regions generated by the detection model (if $p_{i}^{*}$ = 1, the region contains a crack; if $p_{i}^{*}$ = 0, it does not).

The classification loss *L_cls_* focuses on correctly identifying the class of each detected region (e.g., crack, background, or other types of damage) (Equation (3)). As in other YOLO-based approaches, it is typically implemented using categorical cross-entropy [22]:

(3) $$L_{cls}=-\sum_{k=1}^{K}y_{k}^{*}log({\hat{y}}_{k})$$

where $y_{k}^{*}$ is the true class label of the detected region for class *k*, ${\hat{y}}_{k}$= is the predicted probability of the model for class *k*. For instance, if a region is labeled as a crack, then $y_{crack}^{*}=1$; otherwise, this value is set to 0 for other classes.

The total loss function $L_{total}$ (Equation (4)) combines localization, confidence, and classification losses, each weighted by hyperparameters $\lambda_{1}$ and $\lambda_{2}$ [21]:

(4) $$L_{total}=L_{box}+\lambda_{1}L_{conf}+\lambda_{2}L_{cls}$$

The localization loss $L_{box}$ typically uses Euclidean distance or Intersection over Union (IoU) to compare the predicted and ground truth bounding box coordinates. This loss formulation enables the model to jointly optimize for spatial localization, object existence, and class prediction, leading to both accurate detection and classification [23].

In this study, a deep learning-based object detection approach is employed to enable fast and accurate identification of structural damage following earthquakes. The following sections detail the model architecture, training strategy, and performance evaluation, supported by rigorous mathematical formulations.

### 2.2. Model Architecture and Performance Indicators

The purpose of this study is not to provide a benchmark comparison among different object detection architectures but to demonstrate the feasibility of integrating a deep learning-based crack detector into a quadrupedal robotic inspection platform. For this reason, YOLOv8n was selected as a practical model owing to its favorable balance between detection accuracy, inference speed, implementation simplicity, and compatibility with GPU-enabled edge devices such as the NVIDIA Jetson AGX Xavier. Its single-stage and anchor-free architecture enables low-latency detection of surface cracks with different orientations and aspect ratios, which is important for robotic inspection under time-constrained post-earthquake scenarios. Nevertheless, future studies will include comparative evaluations with alternative detectors, such as Faster R-CNN, SSD, YOLOv5/YOLOv7, and transformer-based models, to further assess model-dependent performance differences.

**Figure 6:**
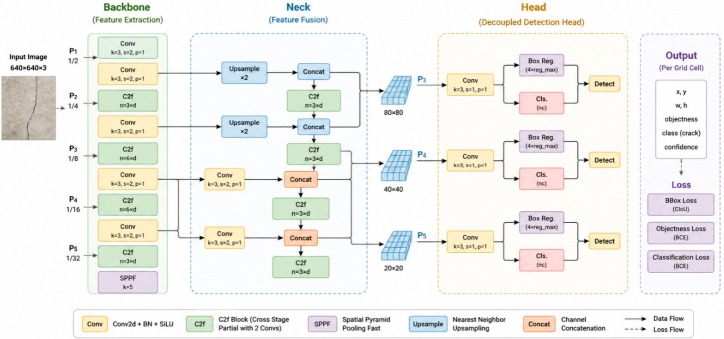
Simplified YOLOv8-based detection architecture used for crack localization.

Although two-stage object detection methods such as R-CNN and its derivatives [22,24] are known for their high accuracy, single-stage architectures have become increasingly popular in real-time applications due to their faster inference speed [23]. In this study, YOLOv8n [25,26] was chosen for its anchor-free design, which allows the model to efficiently detect structural cracks of varying shapes and sizes in a single forward pass (see Figure 6).

The model takes an RGB input image $I\in R^{H\times W\times 3}$ and processes it through a series of convolutional layers (CNN blocks). For each spatial location, the model outputs a bounding box B=(x,y,w,h) and a corresponding class score γ. In this study, the focus is specifically on detecting the crack class. Where (x,y) represent the center coordinates of the box, and (w,h) denote its width and height, respectively.

The dataset used in this study consists of 3255 images, with cracks meticulously annotated in the YOLO format. To improve the model’s adaptability to various real-world conditions, data augmentation techniques were applied, introducing variations in lighting, viewing angles, and surface characteristics such as roughness and low light. These augmentations were intended to enhance the model’s ability to generalize across different damage patterns and environmental conditions [27].

The YOLOv8n model was trained for 100 epochs using the YOLOv8n architecture, which was selected due to its lightweight structure, low computational demand, and suitability for real-time edge deployment. During training, the input images were resized to 640 × 640 pixels, and a batch size of 8 was used to balance detection performance and GPU memory usage. Stochastic Gradient Descent (SGD) with momentum was adopted as the final optimizer [28]. The initial learning rate was set to 0.01 and gradually reduced during training according to the learning-rate scheduling strategy. The total training duration for 100 epochs was 2.293 h, corresponding to approximately 2 h 18 min. At the end of each epoch, key performance metrics, including Precision, Recall, and mean Average Precision (mAP), were computed using a validation set to monitor progress and reduce the risk of overfitting [29]. Both the best-performing and final model weight files were saved and later optimized for the inference stage. The main training parameters used for the crack detection model are summarized in Table 1.

**Figure 7:**
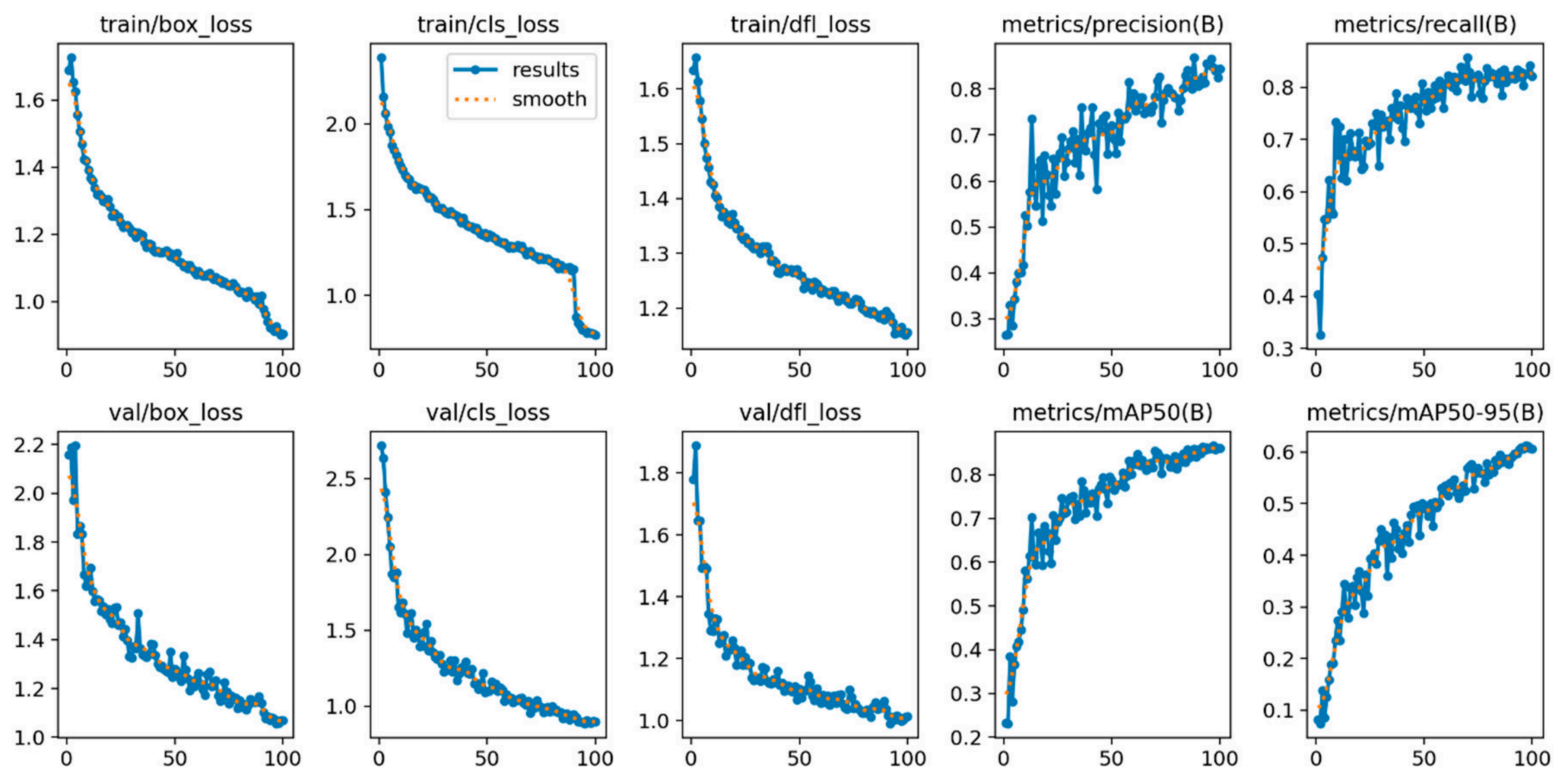
Training and validation curves of the YOLOv8n model over 100 epochs, illustrating loss and key performance metrics.

After completing the training process, the developed model was thoroughly evaluated on an independent test dataset. Precision, recall, and mAP were calculated by comparing the predicted crack bounding boxes with the manually annotated ground-truth boxes. A prediction was considered a true positive when its Intersection over Union (IoU) with the corresponding ground-truth box was equal to or greater than the selected threshold; otherwise, unmatched predictions were counted as false positives and missed ground-truth cracks as false negatives. Precision and recall were calculated as TP/(TP + FP) and TP/(TP + FN), respectively. The mAP@50 value was calculated at IoU = 0.50, while mAP@50–95 was obtained by averaging AP values over IoU thresholds from 0.50 to 0.95. Since this study focuses on a single crack class, the reported mAP values represent crack-class detection performance. The results demonstrated strong performance, with a precision of 84.6%, recall of 82.6%, mAP@50 of 86.7%, and mAP@50–95 of 61.3%, indicating high accuracy and robust generalization for crack-based visual damage detection (Figure 7). With an average inference time of approximately 4.3 milliseconds per image, the model is well-suited for real-time applications, making it particularly useful in on-site scenarios that require rapid decision-making. The evaluation was performed using a single training–validation–test pipeline. Repeated trials and cross-validation were not conducted because the main aim of this study was to validate the integration of YOLOv8n-based crack detection with a quadrupedal robotic inspection platform. This is acknowledged as a limitation, and repeated runs and cross-validation will be considered in future work.

IoU-based analysis showed that the majority of cracks were accurately detected at an IoU threshold of ≥0.5. Even under challenging conditions such as low light or high noise, the model maintained reliable performance, thanks to effective data augmentation and hyperparameter tuning.

**Figure 8:**
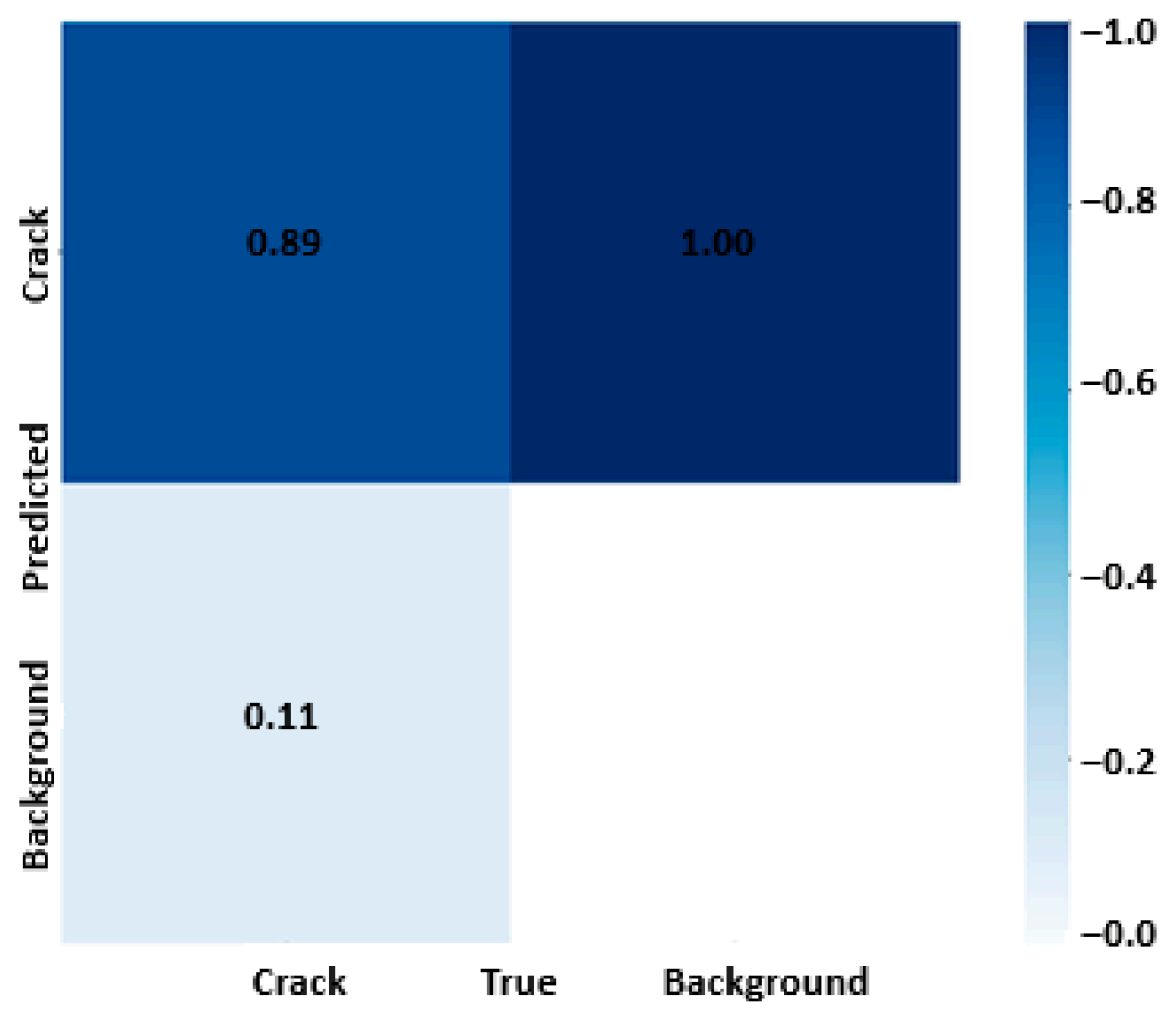
Normalized confusion matrix showing classification performance for crack and background classes on the test dataset.

The normalized confusion matrix in Figure 8 provides further insight into classification performance. The model correctly identified 89% of “Crack” instances, highlighting its effectiveness in detecting structural damage. However, 11% of cracks were misclassified as “Background,” suggesting that some complex or subtle crack patterns may still be difficult to distinguish. In contrast, the model achieved 100% accuracy in identifying the “Background” class, demonstrating its strong ability to differentiate non-damage regions. Overall, the model demonstrates high accuracy and strong generalization in crack-based visual damage detection.

**Figure 9:**
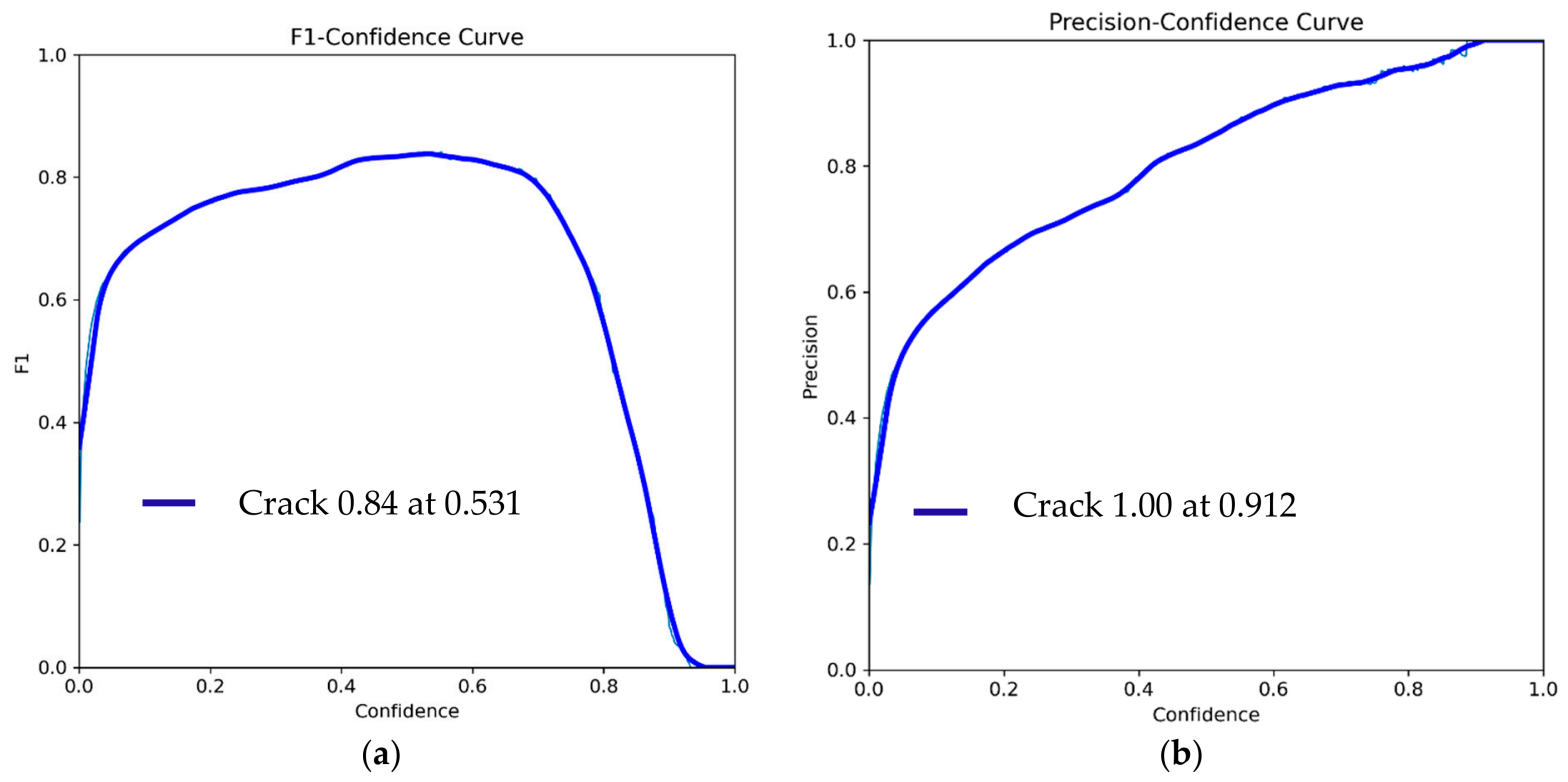
(**a**) F1–confidence curve and (**b**) precision–confidence curve, illustrating the variation in model performance metrics with respect to confidence threshold and supporting optimal threshold selection.

Figure 9a displays the F1-Confidence Curve, which illustrates how the model’s F1 score varies across different confidence thresholds. The F1 score, representing the harmonic mean of precision and recall, serves as a key indicator of overall model performance. This curve allows for an in-depth evaluation of how threshold selection influences accuracy.

The graph shows that the maximum F1 score of 0.84 is achieved at a confidence threshold of 0.531, suggesting that this threshold offers the best balance between precision and recall. At lower confidence levels, the F1 score increases rapidly, but it declines as the threshold becomes too high, indicating that overly strict confidence settings can reduce detection performance by filtering out true positives.

Figure 9b shows how precision varies with the prediction confidence threshold for the crack-based visual damage detection model. Precision, defined as the ratio of true positives to all positive predictions, is essential for evaluating the accuracy of the model’s detections. The curve shows that the model reaches its highest precision of 1.00 at a confidence threshold of 0.912, indicating that all predictions above this level are correct. At lower thresholds, precision declines due to an increase in false positives. As the threshold increases, precision improves steadily until it levels off. This trend highlights the trade-off between sensitivity and specificity, emphasizing the importance of selecting an appropriate confidence threshold, especially in applications where minimizing false positives is critical. Therefore, threshold tuning is recommended to align the model’s performance with the demands of safety-critical, real-time damage assessment scenarios.

**Figure 10:**
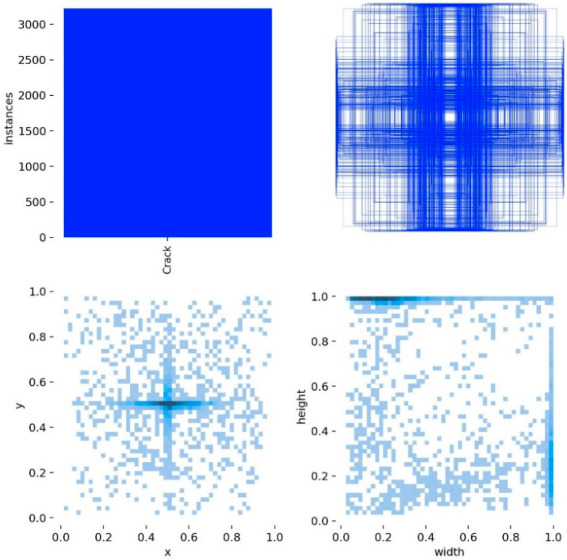
Correlogram of bounding-box parameter relationships.

A correlation matrix was used to examine the pairwise relationships and sample densities among the normalized bounding box attributes (x center, y center, width, and height) for all crack instances in the dataset (see Figure 10). The matrix reveals a clear clustering of bounding box centers near the image midpoint, indicating that cracks tend to appear in central regions. In contrast, the relationships between x center and width, as well as y center and height, show more dispersed patterns, suggesting that crack size is not strongly dependent on position. A moderate correlation between width and height indicates that most cracks exhibit a relatively narrow range of aspect ratios. Color intensity in the matrix represents sample density, with darker regions indicating higher annotation frequency. This visualization provides insights into the spatial distribution of labeled data and can inform the design of data augmentation strategies and adjustments to model configuration.

**Figure 11:**
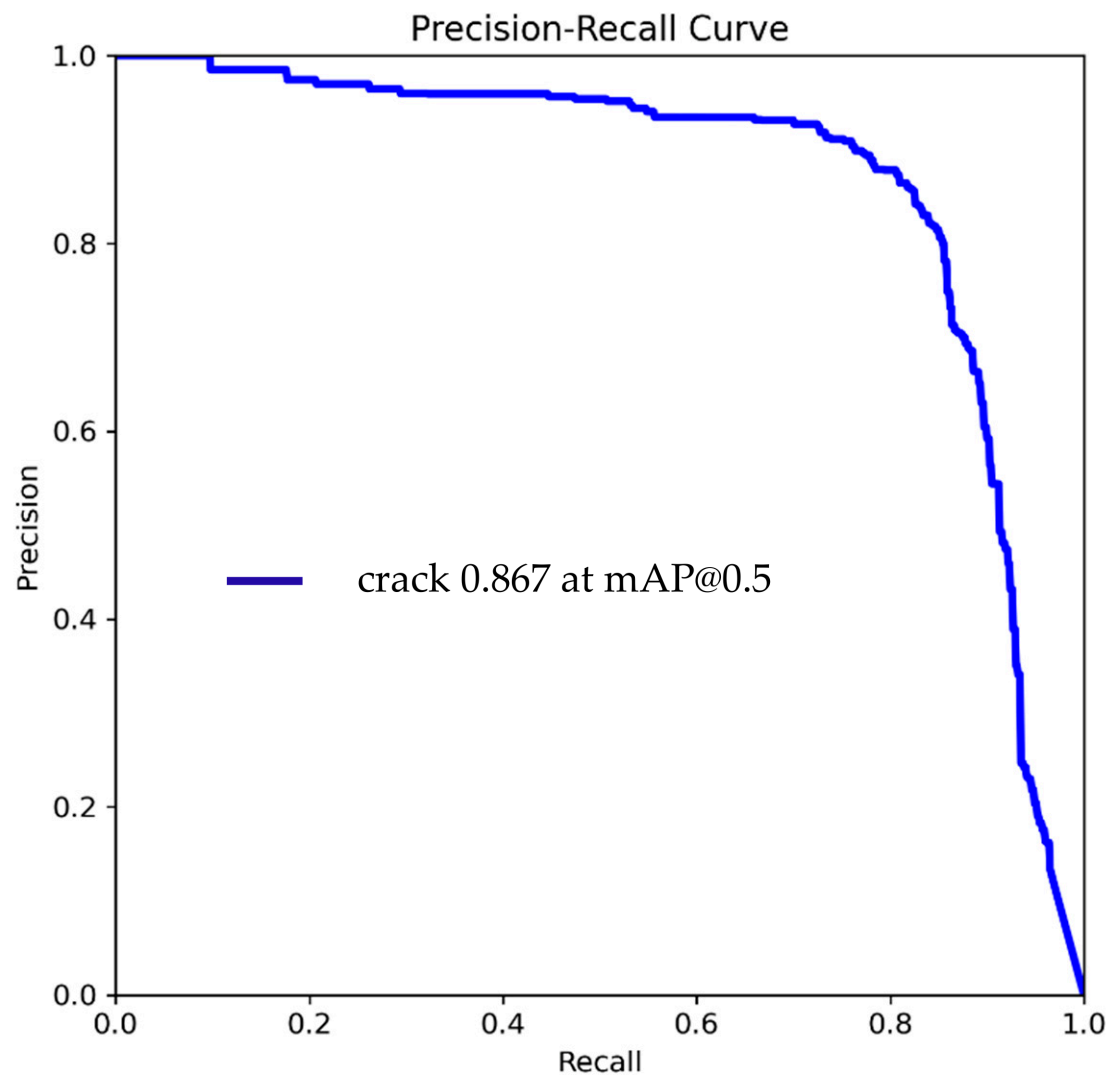
Precision–recall curve.

The precision-recall curve, shown in Figure 11, illustrates the trade-off between precision (the proportion of true positives among all positive predictions) and recall (the proportion of true positives detected among all actual instances) across varying confidence thresholds. For the crack class, the model achieves a maximum precision of 0.867, demonstrating strong accuracy in identifying crack instances. The mean Average Precision at an IoU threshold of 0.5 (mAP@0.5) is also 0.867, indicating reliable overall detection performance. At low recall levels, the curve exhibits an initial plateau, reflecting the model’s ability to maintain high precision with few false positives. As recall increases, precision gradually declines, which is expected as the model becomes more sensitive and potentially includes more false positives. This pattern highlights the inherent trade-off between sensitivity and specificity and underscores the importance of choosing an appropriate confidence threshold to balance the two.

Overall, the precision–recall curve offers valuable insights for optimizing threshold selection. It also reveals opportunities to enhance recall without significantly sacrificing precision—an important consideration for real-time, safety-critical structural damage assessment tasks.

**Figure 12:**
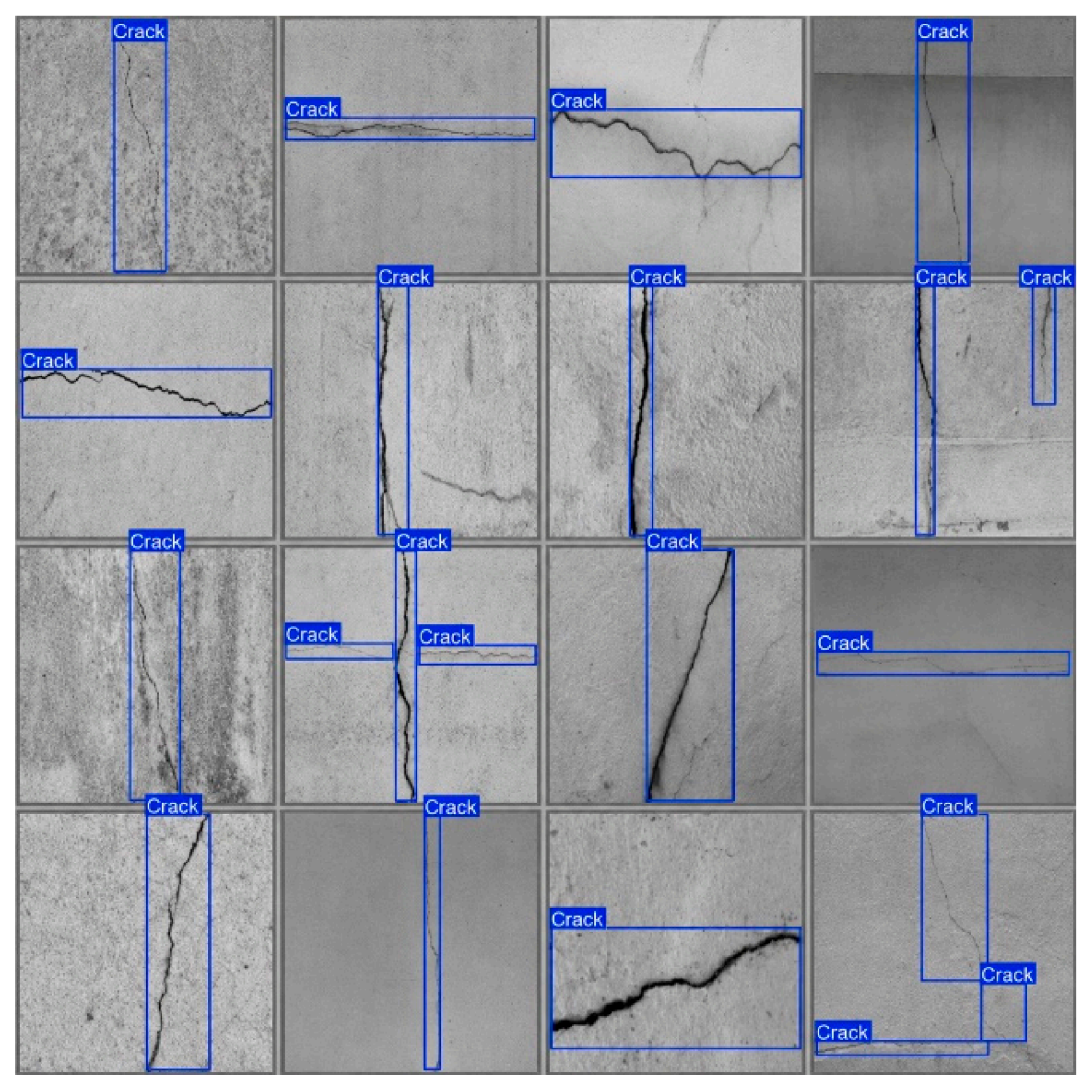

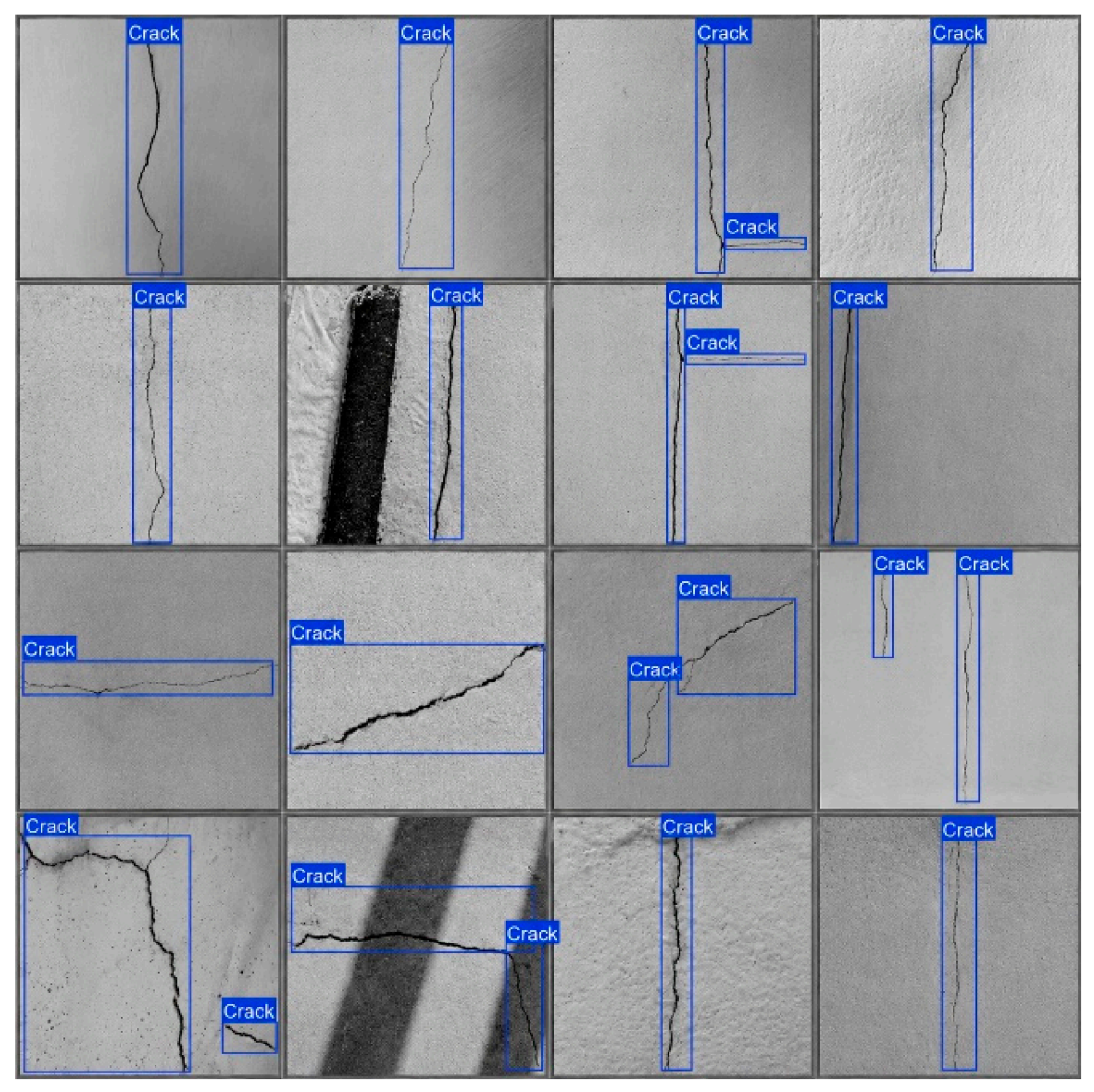
Sample crack detection results with bounding boxes on validation images.

Figure 12 shows the model’s crack-based visual damage detection results on selected validation images. Detected cracks are marked with blue bounding boxes, illustrating the model’s ability to accurately localize and identify cracks across a range of sizes, shapes, and orientations. The consistent and precise placement of these bounding boxes highlights the model’s strong detection performance and ability to generalize to previously unseen examples. These visual results provide qualitative evidence of effective feature learning and confirm the model’s capability to distinguish crack regions from background noise. This reinforces its suitability for real-time structural damage assessment applications.

## 3. Autonomous Robotic System for Crack-Based Visual Damage Detection

This section presents the integration of the Unitree Go2 quadrupedal robot into a ROS 2-based software framework, enabling it to perform deep learning inference for autonomous crack-based visual damage detection. The system combines legged mobility with real-time data acquisition and processing using an Intel RealSense D435i RGB-D camera and a YOLOv8n object detection model.

### 3.1. Embedded Sensing and Processing Components

**Figure 13:**
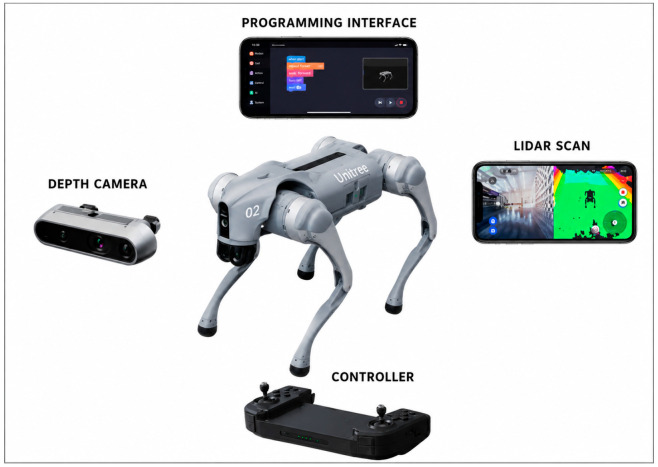
Equipment of Unitree Go2 robot dog.

The Unitree Go2 quadrupedal robot [30], shown in Figure 13, features a four-legged locomotion system designed for stability and mobility on uneven terrain. Its lightweight yet durable chassis supports various sensors and payloads, making it adaptable to different inspection tasks. An Intel RealSense D435i RGB-D camera is mounted on the platform to provide synchronized RGB and depth data, enabling accurate 3D perception of the environment. The robot is equipped with joint angle encoders, an inertial measurement unit (IMU), and internal proprioceptive sensors that deliver continuous state feedback to the control system, ensuring dynamic balance and precise gait control. Thanks to its modular design, the Go2 allows for quick integration of additional sensing or computing components without affecting its locomotion performance.

The robotic control and perception software stack was deployed on Ubuntu 22.04 LTS, with ROS 2 Humble serving as the primary middleware for inter-process communication and system orchestration [31].

ROS 2 was selected as the robotic middleware because it provides a modular and distributed software architecture suitable for integrating heterogeneous robotic components within a single inspection framework. In the proposed system, camera data, depth information, robot state feedback, motion commands, and YOLOv8-based inference outputs must be exchanged between different software modules with low latency and reliable synchronization. ROS 2 supports this requirement through its publisher–subscriber communication model, configurable Quality of Service settings, and compatibility with existing drivers such as realsense-ros and Unitree robot interfaces. Therefore, ROS 2 was preferred to facilitate scalable integration of sensing, perception, navigation, and crack-based visual damage detection modules on the Unitree Go2 robotic platform.

**Figure 14:**
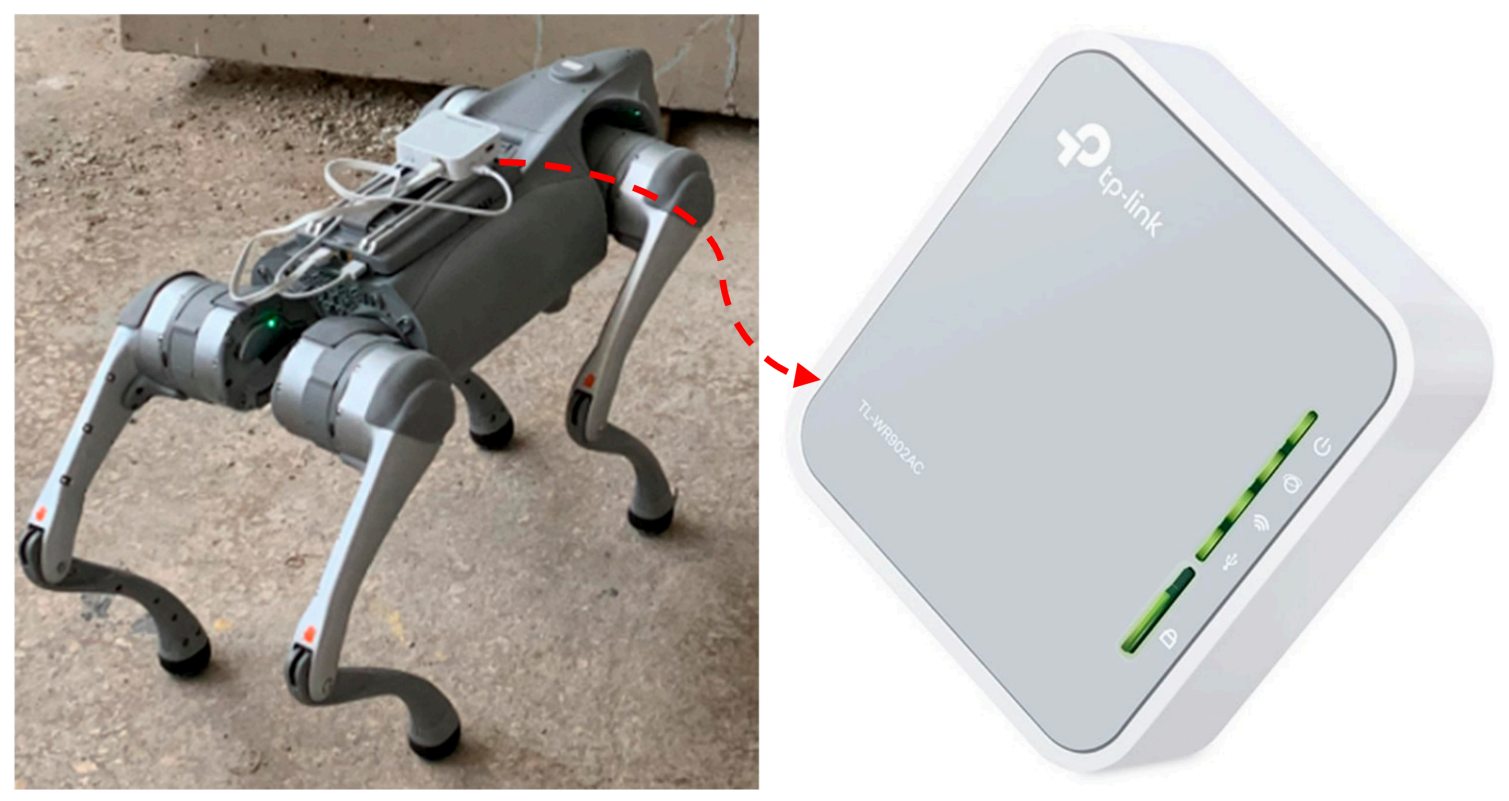
AC750 Wi-Fi repeater.

Sensor drivers—including unitree_ros for the Unitree Go2 platform and realsense-ros for the Intel RealSense D435i camera—are configured as ROS 2 nodes that publish synchronized topics for kinematic state, IMU data, RGB images, and depth maps in real time. Communication between the robot and the host workstation occurs over Ethernet or Wi-Fi (via an AC750 repeater; see Figure 14), ensuring low-latency data transfer and remote monitoring capabilities.

Low-level actuator commands and high-level motion directives are mediated by the Unitree Legged SDK, which provides a C++/Python (Version ≥ 3.8) API for issuing gait, velocity, and posture instructions. This SDK has been seamlessly integrated into the ROS 2 framework to guarantee precise temporal alignment between locomotion commands and sensor data acquisition. Consequently, autonomous navigation, Simultaneous Localization and Mapping (SLAM), and YOLOv8n inference modules operate concurrently within a unified ROS 2 lifecycle, enabling robust, real-time crack-based visual damage detection in complex environments [22].

### 3.2. Motion and Autonomous Navigation

Quadrupedal locomotion control requires coordinated regulation of each leg’s joint trajectories to produce a dynamically stable gait [32]. The Unitree Go2 continuously fuses proprioceptive sensor data—including joint encoder readings, accelerometer, and gyroscope measurements—to estimate its center of mass (CoM) state and maintain balance over uneven terrain. The discrete-time CoM update can be expressed in Equation (5) as:

(5) $$x_{\mathrm{CoM}}(t+\Delta t)=f(q(t),\dot{q}(t),u(t))$$

where q is the vector of joint angles, $\dot{q}$ their angular velocities, and u the control input vector.

### 3.3. Real-Time Integration of Camera Data and Deep Learning Inference

The Intel RealSense D435i camera streams color (RGB) and depth data as separate ROS 2 topics at 30 Hz. These frames are preprocessed before being passed to the YOLOv8n object detector, including resizing to match the model’s input resolution, pixel value normalization, and format conversion [21]. Depth data is used to calculate the three-dimensional position of detected damage instances, such as the height of a crack relative to the floor. In the current implementation, the detected crack regions are localized at the image level using YOLOv8 bounding boxes and confidence scores. The system does not yet perform metric coordinate transformation or georeferenced damage mapping. Instead, the detected crack images are automatically recorded and presented to the user/operator with visual annotations after the robotic inspection. Therefore, the present study focuses on image-based crack detection and visual reporting rather than global coordinate-based damage localization.

Real-time inference was performed onboard using the NVIDIA Jetson AGX Xavier, where GPU acceleration enabled an average YOLOv8n model-level inference latency of 4.3 ms per frame after image preprocessing. This value should not be interpreted as the full end-to-end latency of the robotic inspection pipeline. The complete online workflow also includes RGB-D frame acquisition from the Intel RealSense D435i camera, ROS 2 message transfer, image resizing and normalization, post-processing of bounding boxes and confidence scores, and publication of detection results within the ROS 2 framework. During the laboratory experiments, the system processed camera frames online at the acquisition rate used in the tests. However, detailed component-wise latency and embedded-system indicators, such as GPU utilization, memory usage, power consumption, thermal behavior, and battery-runtime impact, were not continuously logged. Since these factors are critical for field deployment, future work will include resource-utilization and end-to-end latency benchmarking under different image resolutions, frame rates, communication modes, navigation speeds, and environmental conditions.

## 4. Laboratory Validation of Robotic Crack-Based Visual Damage Detection System

**Figure 15:**
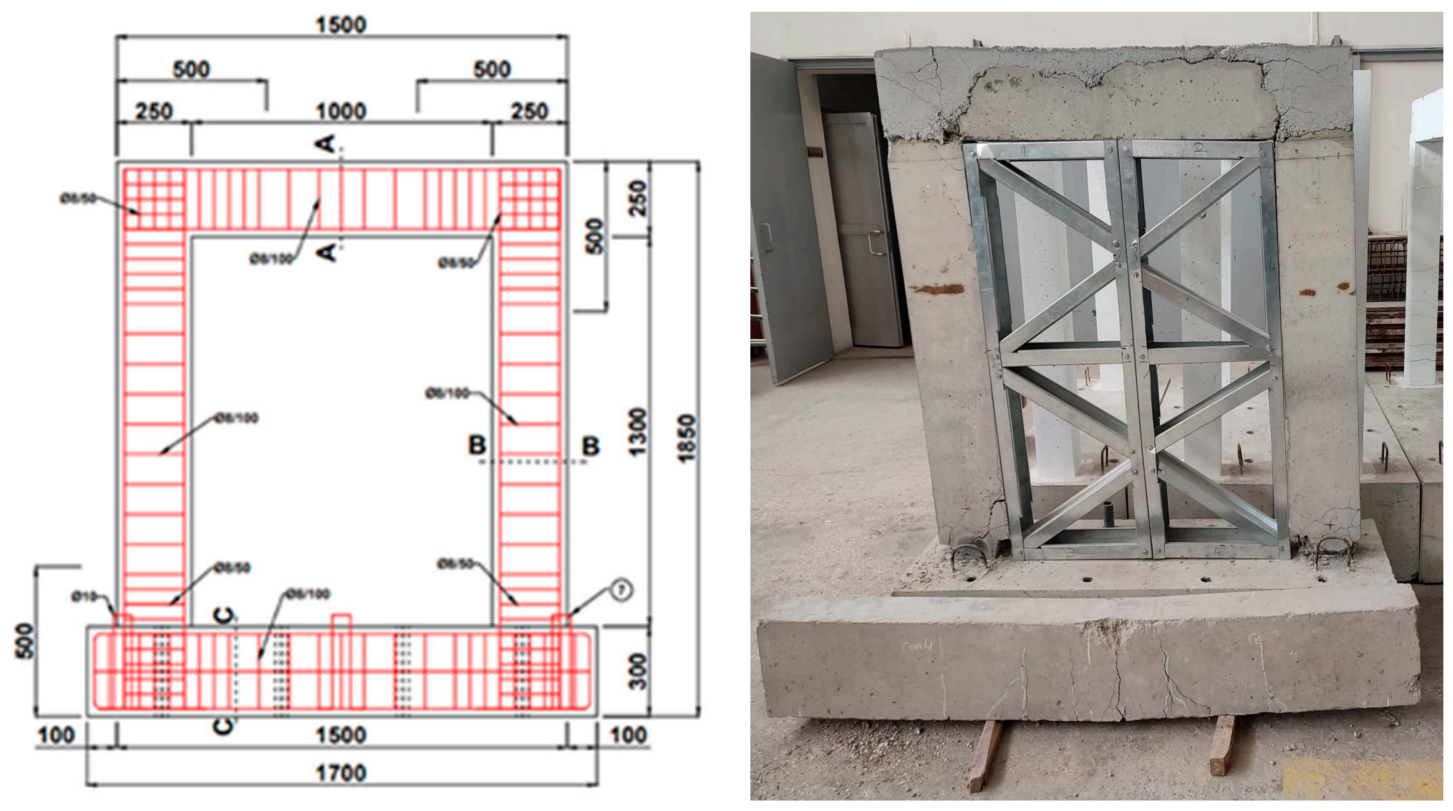
RC specimen constructed in the laboratory and its post-damage condition.

The effectiveness of the YOLOv8-based damage detection model was assessed through a series of controlled laboratory experiments. The integration between the Unitree Go2 quadrupedal robot and the deep learning inference pipeline was first verified to ensure seamless real-time data acquisition and processing. The crack-based visual damage detection was carried out using a custom-built reinforced concrete frame specimen designed to simulate realistic post-earthquake damage scenarios. Buildings may show signs of relevant cracks (such as shear and bending cracks and spalling) after an earthquake. The test specimen was constructed using C25/30-grade concrete and B420C reinforcing steel, with dimensions of 550 mm (width) × 1700 mm (length) × 300 mm (depth), and a total height of 1850 mm (Figure 15). Refer to the research by Çolakoğlu et al. [33] for information on the test sample, test procedure and damage cases.

During testing, the Unitree Go2 executed pre-programmed gait patterns to systematically scan the specimen’s surfaces. The onboard Intel RealSense D435i RGB-D camera captured synchronized color and depth images at 30 Hz, which were processed in real time by the YOLOv8n model running on an NVIDIA Jetson AGX Xavier. Detected bounding boxes were recorded and compared against manually annotated ground truth labels to compute key performance metrics.

Results from the laboratory experiments showed high detection performance, with precision, recall, and mean Average Precision (mAP@50) all exceeding 85%. The inference latency remained under 5 milliseconds per frame, confirming the system’s capability for real-time operation. These findings validate the proposed robotic and deep learning integration as a reliable and efficient solution for automated crack detection in reinforced concrete structures under controlled conditions.

**Figure 16:**
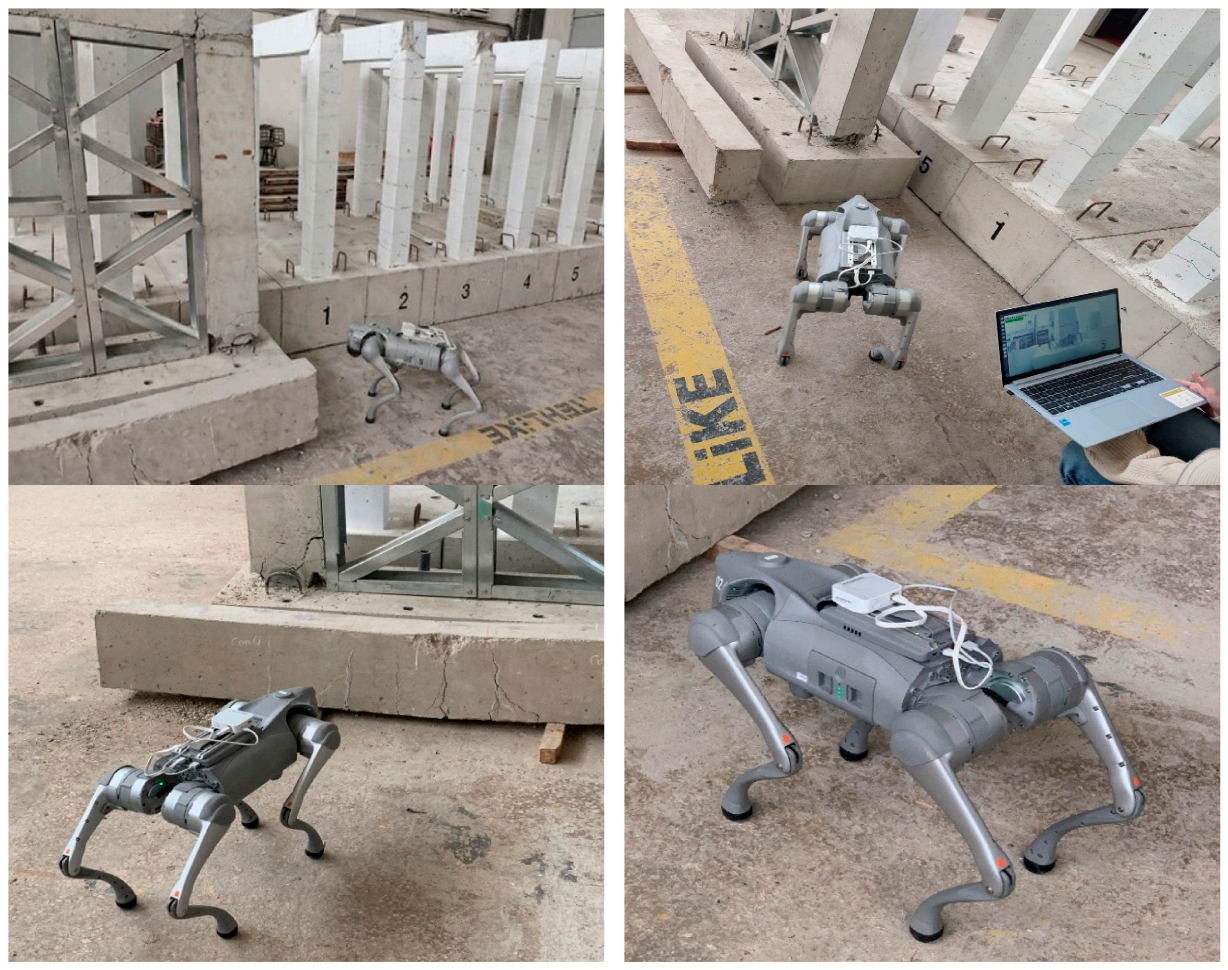
Remote operation of the Unitree Go2 robot during crack-based visual damage detection of the RC specimen.

The measurement procedure was carried out with strict adherence to protocol and oversight. The Unitree Go2 platform was remotely operated (see Figure 16) to systematically traverse all accessible surfaces of the test specimen. During this process, the onboard Intel RealSense D435i camera continuously streamed real-time RGB-D imagery to a ground station workstation, allowing for ongoing visual inspection and documentation of the reinforced concrete frame. Each side of the specimen was thoroughly scanned for surface discontinuities, with all visible cracks identified, annotated, and recorded for subsequent quantitative analysis.

**Figure 17:**
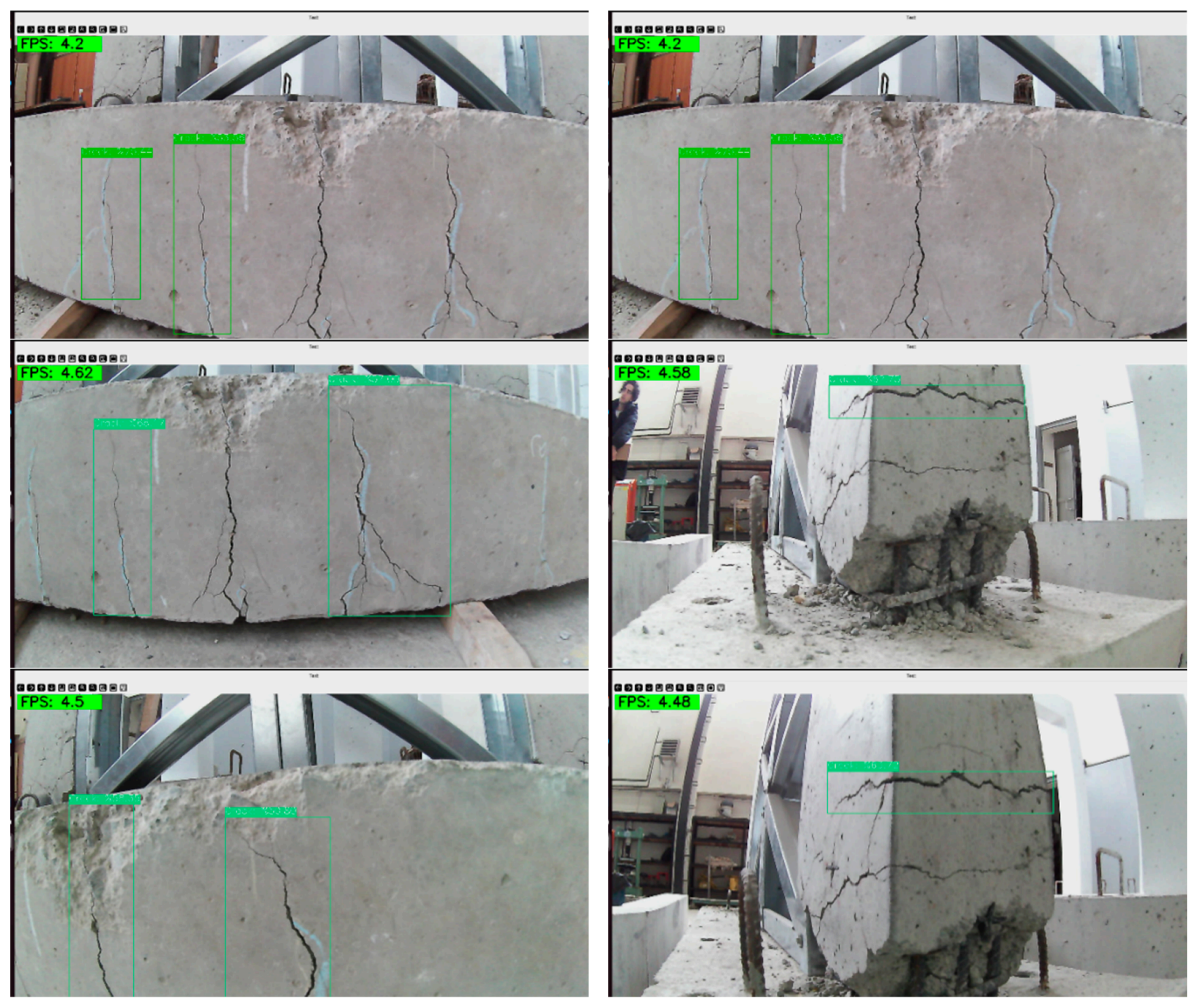

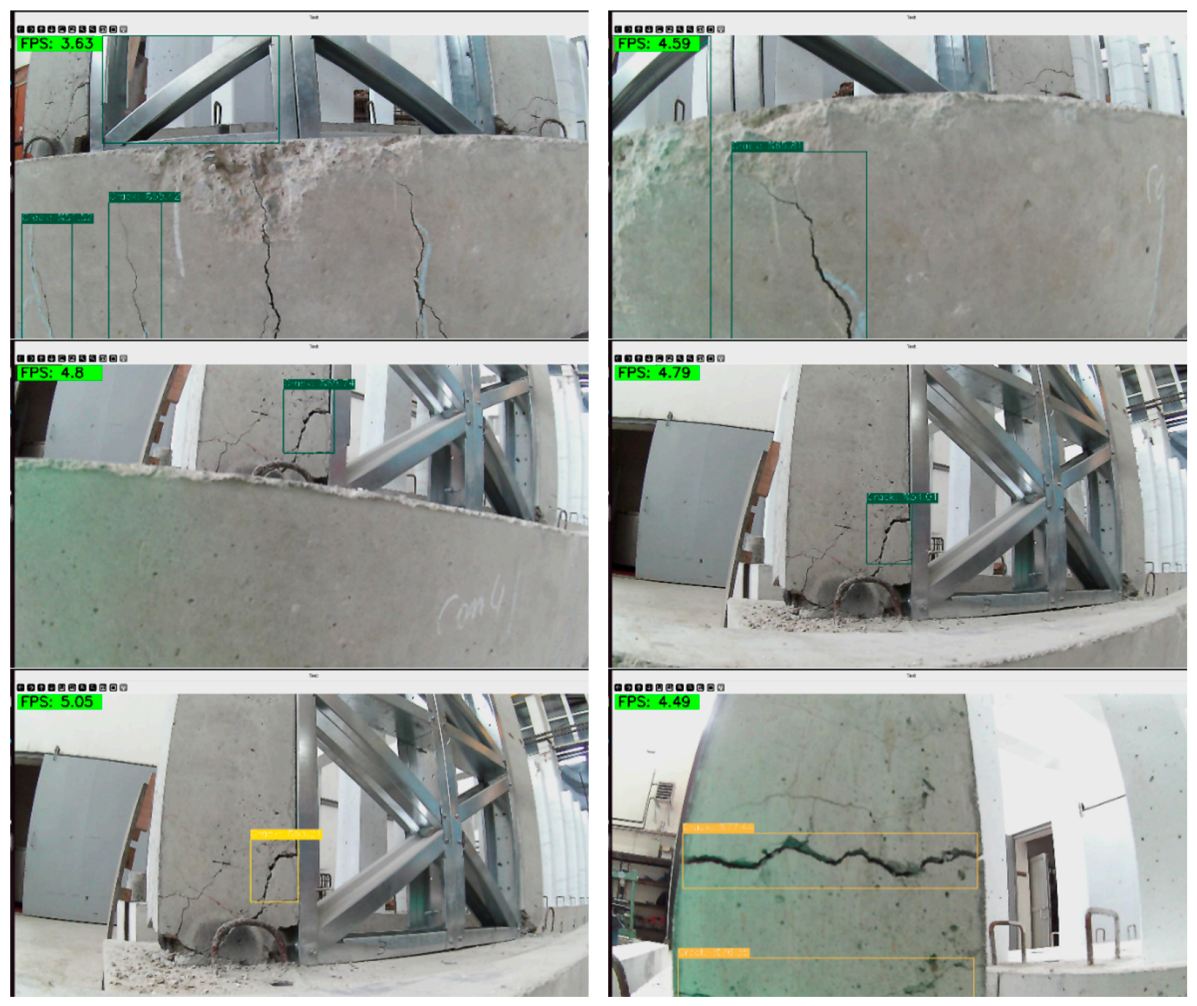
YOLOv8n-based crack detection results on the RC specimen, showing bounding box localization under laboratory conditions.

During laboratory trials, the YOLOv8n crack-based visual damage detection model demonstrated strong performance in accurately identifying and localizing surface cracks. As shown in Figure 17, qualitative analysis of the model’s output revealed that bounding boxes were closely aligned with actual crack boundaries, effectively capturing their position and extent. The model demonstrated high detection performance under controlled laboratory conditions, with bounding boxes generally well-aligned with crack regions; however, some false positives and false negatives were observed, consistent with the reported precision and recall values. These results highlight enhanced YOLOv8n architecture as a reliable solution for structural health monitoring, capable of delivering high-precision damage detection in real-world scenarios. Additionally, the seamless integration of the robot’s real-time image acquisition system with the deep learning inference engine validated the end-to-end inspection pipeline, demonstrating both operational effectiveness and readiness for field deployment.

## 5. Discussion

The results of this study demonstrate the feasibility of integrating deep learning-based crack detection with a quadrupedal robotic inspection platform for post-earthquake visual assessment. The YOLOv8n model achieved high crack detection performance under controlled laboratory conditions, with precision, recall, and mAP@50 values exceeding 85%. These results indicate that the trained detector can effectively localize visible surface cracks on reinforced concrete specimens. However, the observed false positives and false negatives also show that the detection of fine, low-contrast, partially occluded, or irregular crack patterns remains challenging. Therefore, the reported performance should be interpreted as a controlled laboratory validation of crack-based visual detection rather than as a complete structural safety assessment.

Compared with conventional image-based crack detection studies, the main contribution of this work is not limited to model training. Instead, the study demonstrates the integration of crack detection into a robotic inspection workflow involving the Unitree Go2 quadrupedal robot, RGB-D image acquisition, onboard edge computing, and ROS 2-based system communication. Many previous deep learning-based studies focus primarily on image datasets and offline model evaluation, whereas the present study evaluates the trained detector within a robotic inspection scenario. This integration is important because post-earthquake inspection requires not only accurate image classification or localization but also safe data acquisition in potentially hazardous environments.

In comparison with UAV-based post-earthquake inspection systems, the proposed quadrupedal platform offers a different operational advantage. UAVs are highly useful for rapid exterior surveying, roof inspection, and large-area damage screening. However, their operation may be limited in indoor, confined, debris-laden, or GPS-denied environments. A quadrupedal robot can provide closer ground-level inspection and can potentially access areas where human inspectors may face safety risks. Therefore, the proposed system should be considered complementary to UAV-based inspection rather than a replacement for aerial platforms.

The laboratory validation confirmed that the robotic platform can acquire images and perform crack detection during inspection. Nevertheless, several practical limitations remain. The current implementation focuses on visible surface crack detection and does not directly distinguish between cosmetic cracks and structurally critical damage. Such interpretation requires additional engineering assessment and contextual information about the structural element, crack pattern, crack width, crack depth, deformation state, and load-bearing relevance. Similarly, quantitative crack-width measurement, metric coordinate transformation, SLAM-based localization, and georeferenced crack mapping were not implemented in the present study. These aspects are therefore identified as future developments.

The reported inference time of 4.3 ms represents the model-level YOLOv8n inference latency. Although the complete robotic pipeline operated online during laboratory testing, end-to-end latency may vary depending on camera acquisition, ROS 2 communication, preprocessing, post-processing, and robot operation conditions. For real post-earthquake deployment, additional testing is required under dust, debris, variable illumination, surface texture changes, unstable structural conditions, and communication constraints. Future work should therefore include field-like validation, repeated training trials, cross-validation, comparative benchmarking with alternative object detection models, and extension of the system toward multi-damage detection, crack-width estimation, and SLAM-supported damage mapping.

Overall, the findings suggest that combining quadrupedal robotics with deep learning-based crack detection can support rapid preliminary visual inspection after earthquakes. The proposed system has the potential to reduce human exposure to hazardous environments and assist engineers by automatically identifying and presenting crack regions for further evaluation. However, the current system should be interpreted as a robotic visual inspection aid, not as a standalone decision-making tool for structural safety classification.

## 6. Conclusions and Recommendations

This study presented a quadruped-based robotic inspection system for crack-based visual damage detection in post-earthquake assessment scenarios. The proposed system integrates a Unitree Go2 quadrupedal robot, an Intel RealSense D435i RGB-D camera, an NVIDIA Jetson AGX Xavier edge computing unit, and a YOLOv8n-based object detection model. The main objective was to demonstrate the feasibility of combining robotic mobility, onboard image acquisition, and deep learning-based crack detection within an online laboratory inspection workflow.

- The single-stage and anchor-free YOLOv8n architecture provided an efficient solution for detecting visible surface cracks on reinforced concrete specimens. The main findings of the study are summarized as follows:
- The YOLOv8n detector achieved an average model-level inference latency of 4.3 ms per frame. The complete robotic inspection pipeline operated online during laboratory experiments; however, the end-to-end latency may vary depending on image acquisition, ROS 2 communication, preprocessing, post-processing, and robot operation conditions.
- A dataset comprising 3255 annotated crack images was used for model training and validation. The dataset included images obtained from both field investigations and the Roboflow platform. Independent laboratory tests conducted on reinforced concrete specimens demonstrated high crack detection performance, with precision, recall, and mAP@50 values exceeding 85%.
- The model showed effective detection capability for visible surface cracks with different orientations and appearances under controlled laboratory conditions. However, some false positives and false negatives were observed, indicating the need for further improvement in detecting fine, low-contrast, or complex crack patterns.
- The integration of image acquisition, onboard deep learning inference, and robotic inspection was successfully demonstrated using a reinforced concrete laboratory specimen. The system automatically detected crack regions in the captured images and presented annotated visual outputs, including bounding boxes and confidence scores, to the user/operator after inspection.
- In the present implementation, the system performs image-based crack detection and visual reporting. It does not yet perform direct physical crack-width measurement, metric coordinate transformation, SLAM-based localization, or georeferenced crack mapping. Therefore, the current system should be considered a laboratory-validated robotic visual inspection framework rather than a fully autonomous structural diagnosis or mapping system. More specifically, it should be interpreted as a robotic visual inspection aid for detecting visible surface cracks, not as a standalone tool for determining structural safety or distinguishing cosmetic defects from structurally critical damage.
- The results indicate that quadrupedal robotics combined with deep learning can provide a promising tool for reducing human exposure during post-earthquake inspection tasks and supporting rapid preliminary visual assessment of damaged structures.

To build upon the findings of this study, future research will focus on the following directions:

- The system will be tested in real post-earthquake or field-like environments to evaluate its robustness under variable lighting, surface texture, dust, debris, access limitations, and unstable structural conditions.
- Additional data augmentation strategies, more balanced datasets, and expanded training data will be used to improve the detection of fine, micro-scale, low-contrast, and partially occluded cracks.
- The detection capability will be extended beyond surface cracks to include other visible damage types such as spalling, delamination, corrosion-related deterioration, exposed reinforcement, and material loss.
- Quantitative crack-width estimation will be incorporated using calibrated RGB-D imagery, pixel-to-metric conversion, and segmentation- or edge-based measurement techniques.
- Advanced mapping and localization methods, including SLAM, robot-pose-based localization, camera-to-robot coordinate transformation, and georeferenced crack mapping, will be investigated in future implementations.
- Future studies will also include repeated training trials, cross-validation, and comparative benchmarking with alternative object detection models to evaluate the statistical robustness and model-dependent performance of the proposed approach.
- More advanced autonomous navigation and path-planning strategies will be explored to improve inspection capability in cluttered, partially collapsed, or hazardous post-earthquake environments.
- Future work will include detailed embedded-system benchmarking on the NVIDIA Jetson AGX Xavier, including GPU utilization, memory usage, power consumption, thermal behavior, and battery-runtime analysis during online robotic inspection.

## Figures and Tables

**Table 1:** Main training parameters used for the YOLOv8n crack detection model.

Parameter	Value
Detection framework	YOLOv8n
Detection task	Single-class surface crack detection
Annotation format	YOLO bounding-box format
Input image size	640 × 640 pixels
Training images	2658
Validation images	300
Independent test images	297
Number of epochs	100
Batch size	8
Initial learning rate	0.01
Optimizer	SGD with momentum
Momentum	0.937
Weight decay	0.0005
Training duration	2.293 h, approximately 2 h 18 min
Edge deployment platform	NVIDIA Jetson AGX Xavier

## Data Availability

The data that support the findings of this study are available from the corresponding author upon reasonable request.
